# Construction of Multiple Switchable Sensors and Logic Gates Based on Carboxylated Multi-Walled Carbon Nanotubes/Poly(*N*,*N*-Diethylacrylamide)

**DOI:** 10.3390/s18103358

**Published:** 2018-10-08

**Authors:** Xuemei Wu, Xiaoqing Bai, Yang Ma, Jie Wei, Juan Peng, Keren Shi, Huiqin Yao

**Affiliations:** 1 College of Pharmacy, Ningxia Medical University, Yinchuan 750004, China; Xuemei_WU157@163.com (X.W.); mayang_182@sina.com (Y.M.); weijie_137@sina.com (J.W.); 2 School of Basic Medical Sciences, Ningxia Medical University, Yinchuan 750004, China; xiaoqing_bai137@sina.com; 3 School of Chemistry and Chemical Engineering, Ningxia University, Yinchuan 750021, China; pengjuan@nxu.edu.cn; 4 State Key Laboratory of High-Efficiency Coal Utilization and Green Chemical Engineering, Ningxia University, Yinchuan 750021, China; shikeren@163.com

**Keywords:** electrocatalysis, encoder, logic gate, matrine, sophoridine

## Abstract

In this work, binary hydrogel films based on carboxylated multi-walled carbon nanotubes/poly(*N*,*N*-diethylacrylamide) (c-MWCNTs/PDEA) were successfully polymerized and assembled on a glassy carbon (GC) electrode surface. The electroactive drug probes matrine and sophoridine in solution showed reversible thermal-, salt-, methanol- and pH-responsive switchable cyclic voltammetric (CV) behaviors at the film electrodes. The control experiments showed that the pH-responsive property of the system could be ascribed to the drug components of the solutions, whereas the thermal-, salt- and methanol-sensitive behaviors were attributed to the PDEA constituent of the films. The CV signals particularly, of matrine and sophoridine were significantly amplified by the electrocatalysis of c-MWCNTs in the films at 1.02 V and 0.91 V, respectively. Moreover, the addition of esterase, urease, ethyl butyrate, and urea to the solution also changed the pH of the system, and produced similar CV peaks as with dilution by HCl or NaOH. Based on these experiments, a 6-input/5-output logic gate system and 2-to-1 encoder were successfully constructed. The present system may lead to the development of novel types of molecular computing systems.

## 1. Introduction

Molecular logic gates can carry out different computations at the molecular level, and the working mechanism or principle is similar to silicon processors [1,2,3,4]. Molecular logic gates, a type of logic gates system, can use biological materials or biomolecules such as enzymes, DNA, or proteins to establish basic or -simple molecular devices and equipment [3,4,5,6,7]. For example, Liu’s group successfully established 4-input/7-output biomolecular logic gates, and provided a new method to construct complex biomolecular logic gate system based on bio-electrocatalysis of natural DNA [8]. An adjustable electrocatalysis was constructed on the basis of multiple responsive interface poly (*N*-isopropylacrylamide co-*N*,*N*’-dimethylaminoethylmethacrylate) (P(NiPAAm-DMEM) films while the immobilization of glucose oxidase (GOD) and horseradish peroxidase (HRP) on the surface of pyrolytic graphite (PG) electrodes was also used to construct logic gates by Liu and coworkers [9]. Because of their huge potential in different areas, including disease diagnosis, sensing, and drug release [10,11,12], the molecular logic gate system has attracted ever increasing attention.

In previous reports, the widely used probe compounds were ferrocene and its derivatives including ferrocene carboxylic acid, ferrocenylmethanol, ferrocene dicarboxylate, potassium ferrocyanide, and hydroquinone [1,13,14,15,16]. For example, Hu’s group [17] studied the switchable behaviors of poly(*N*-isopropylacrylamide) (PNIPAA)-GOD film using ferrocene carboxylic acid as an electroactive probe. The permeability of the reduced graphene oxide/poly(*N*-isopropylacrylamide) (rGO/PNIPAA) dual structure film was investigated by using ferrocene dicarboxylate as a molecular probe, and established a 4-input enabled OR (EnOR) logic gate system [18]. However, drug molecules are becoming increasingly popular probes in the field of electrochemistry and medical research, because some drug molecules with electroactivity can be used to investigate the properties of film systems in vitro or used as target drugs to treat diseases in vivo. Matrine and sophoridine are quinolizidine alkaloids isolated from traditional Chinese medicine such as Sophora flavescens, Sophora subprostrata, or Sophora alopecuroides, which are tetracyclic quinolizine compounds with a molecular skeleton and can be regarded as a combination of two quinolizine rings [19,20]. In recent years, it has been discovered that the alkaloids of S. flavescens exhibit anti-arrhythmic, anti-inflammatory, anti-fibrosis, anti-tumor, and numerous other significant pharmacological activities, and various formulations have been widely used in clinical practice [21,22,23,24]. Therefore, in the present work, we used matrine and sophoridine as target drug molecule probes to study the switching properties of the binary carboxylated multi-walled carbon nanotubes/poly(*N*,*N*-diethylacrylamide) (c-MWCNTs/PDEA) film system. The electrochemical signals of matrine and sophoridine were amplified by means of electrocatalysis, and the 6-input/5-output logic gate systems were constructed. Currently, the logic gate system has become a research hotspot [3] and its applications for clinical drugs are expected in future clinical diagnosis and treatment research.

PDEA hydrogel films were synthesized on glassy carbon (GC) electrodes by cross-linking radical polymerization in this system. PDEA is a widely investigated sensitive polymer, its temperature and salt sensitivity have been reported in previous literature [25,26,27]. However, its methanol sensitivity, especially combined with its temperature and sulfate concentration sensitive on–off property has never been reported by the cyclic voltammetry (CV) method. This inspired us to increase the complexity of the logical gate system by increasing the number of outside stimuli. In the present work, the electroactive drug molecules, matrine and sophoridine, showed CV on–off behaviors at c-MWCNTs/PDEA-film electrodes upon external stimuli of pH, temperature, sulfate concentration, and methanol proportion. A logic gate system and 2-to-1 encoder were further constructed. This may open up the possibility of applying electroactive drug molecular probes in electrochemical biosensors or other devices based on multiple responsive electrocatalysis.

## 2. Experimental Section

### 2.1. Reagents

*N*,*N*’-methylenethyl butyrateisacrylamide (BIS), urease (E.C. 3.5.1.5, type III, 39, 290 units g^−1^), esterase (17,000 units g^−1^), and *N*,*N*,*N*’,*N*’-tetramethylenediamine (TEMED) were obtained from Sigma Aldrich (Beijing, China) and used without further purification. Matrine and sophoridine were purchased from Ningxia Zijinghua Pharmaceutical Co., Ltd. (Yinchuan, China). Multiwalled carbon nanotubes (MWCNTs) were obtained from Shenzhen Nanotech Port Co. Ltd. (Shenzhen, China). *N,N*-diethylacrylamide (DEA) was purchased from TCI (Shanghai, China). Sodium persulfate (Na_2_S_2_O_8_) was purchased from Aladdin (Shanghai, China). Sodium sulfate (Na_2_SO_4_), sodium nitrate (NaNO_3_), potassium nitrate (KNO_3_), sodium chloride (NaCl), and magnesium sulfate (MgSO_4_) were purchased from Tianjin Haiguang Chemical Plant (Tianjin, China). All of the other reagents were of analytical grade. All solutions contained 0.15 M NaCl, and the pH was adjusted using dilute HCl or NaOH. All solutions were prepared with water purified twice by ion exchange and distillation.

### 2.2. Preparation of Poly(N,N-Diethylacrylamide) Hydrogel Films

The typical pre-gel solution containing 0.4 mg mL^−1^ Na_2_S_2_O_8_ initiator, 1.5 mg mL^−1^ BIS cross-linker, 0.46 mg mL^−1^ TEMED accelerator, and 0.5 M DEA monomer, was freshly prepared every time and the air removed with N_2_ before being cast. After about 8 min, the PDEA gel was formed on the surface of the glassy carbon (GC) electrode. During the whole polymerization process, the N_2_ atmosphere was maintained in the bottle. The formed PDEA-film electrode was then immersed in water for 20 min to remove the unreacted reagents.

### 2.3. Apparatus and Procedures

A CHI 660E electrochemical workstation (CH Instruments, Beijing, China) was used for electrochemical measurements using a typical three-electrode cell with a platinum flake as the counter electrode, a saturated calomel electrode as the reference, and the GC electrode with films as the working electrode.

The pH measurements were performed using a PHS-3C pH meter (Shanghai Precision & Scientific Instruments, Shanghai, China). The temperature of solutions in the cell was controlled by a DK-S14 thermostatic bath (Shanghai Jinghong Experimental Equipment Co. Ltd., Shanghai, China) with the precision of ±0.5 °C. The Fourier-transform infrared (FTIR) spectra were recorded at a resolution of 4 cm^−1^ with a Spectrum Two FTIR spectrometer (Perkin Elmer, Shanghai, China). The secondary distilled water used in the experiment was purified by the SZ-97 Automatic Pure water distiller (Shanghai Yarong Biochemical Instruments Co. Ltd., Shanghai, China). The thickness of PDEA films was estimated with a Stereo Discovery V12 stereomicroscope equipped with an AxioCam digital camera (Zeiss) (Tongzhoutongde (Beijing) instrument and meter co. LTD, China). The surface scanning electron microscopy (SEM) of the films was obtained with a JSM 7500F scanning electron microscope (JEOL, Tokyo, Japan) at an acceleration voltage of 10.0 kV. After being treated with different pHs, temperature, Na_2_SO_4_ and methanol, the GC disks were immediately placed into liquid nitrogen to freeze the composite film structure. Afterward, they were placed in an ALPHA 1-2 lyophilizer (Beijing Bomaihang Equipment Co. Ltd., Beijing, China) for 24 h to remove all of the water from the samples. Before SEM imaging, the sample surface was coated with a thin gold film with an SBC-12 sputtering coater (KYKY Technology Development Ltd., Beijing, China). The surface morphology of c-MWCNTs was investigated via transmission electron microscopy (TEM, H-7650 Hitachi, Tokyo, Japan).

## 3. Results

### 3.1. Characterization of Poly(N,N-Diethylacrylamide) (PDEA) Films

Poly(*N*,*N*-diethylacrylamide) (PDEA) hydrogel films were polymerized on a GC electrode by radical cross-linking mainly according to previous reports in the literature [25,28] with certain modifications (as described in Section 2.2). A comparison with the commercial pure DEA sample by IR spectroscopy confirmed the formation of PDEA (Figure S1). The characteristic C=C stretching band at 1610 cm^−1^ observed for DEA monomers was not detected for PDEA, indicating that the DEA monomers were polymerized to PDEA, which was similar to the literature findings [28].

**Figure 1:**
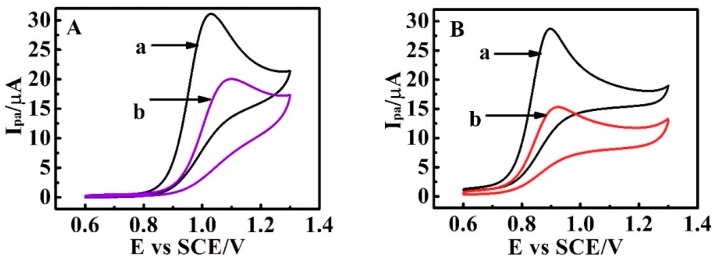
Cyclic voltammetry responses (CVs) of 0.010 M (**A**) matrine and (**B**) sophoridine at 0.05 V s^−1^ and 25 °C in NaCl supporting electrolyte solutions at (a) bare glassy carbon (GC) electrode and (b) poly(*N*,*N*-diethylacrylamide (PDEA) film electrodes.

Matrine, an electroactive drug, was used to characterize PDEA film electrodes through cyclic voltammetry (CV) experiments. An obvious oxidation peak at around 1.02 V, which was the same as the literature [29], was observed at the bare GC electrodes due to electrochemical oxidation of matrine (Figure 1A, curve a). For PDEA film electrodes, the electrochemical oxidation peak current (*I*_pa_) of matrine slightly decreased, and the peak potential shifted positively because the formation of the films hindered the drug from reaching the electrode surface to a certain degree (Figure 1A, curve b), indicating that PDEA hydrogel films had been successfully constructed on the surface of the GC electrode. Sophoridine is an isomer of matrine [30], since they have similar chemical structures (Figure S2). As shown in Figure 1B, sophoridine showed a similar electrochemical behavior to matrine. The electrochemical oxidation peak of sophoridine appeared at about 0.91 V, implying that sophoridine could also be used as an electroactive drug molecule to investigate the switching properties of films. In the following studies, matrine and sophoridine were used as typical molecule probes to study the multiple stimuli-responsive binary films.

### 3.2. pH Responsive Behaviors of Poly(N,N-Diethylacrylamide) Films

**Figure 2:**
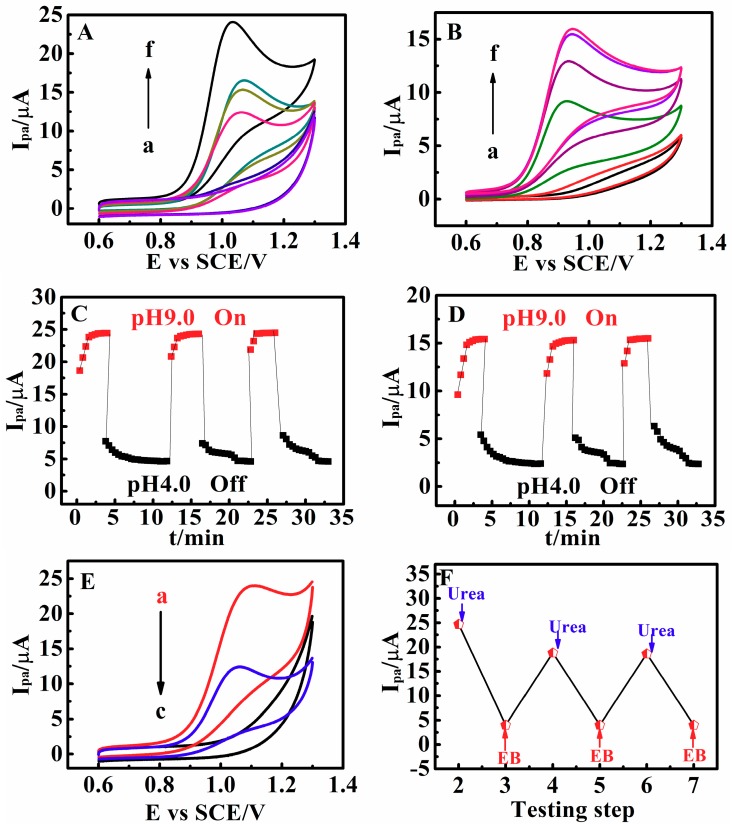
CVs of 0.010 M (**A**) matrine and (**B**) sophoridine at 0.05 V s^–1^ and 25 °C for PDEA films in NaCl solutions at pH (a) 4.0, (b) 5.0, (c) 6.0, (**d**) 7.0, (e) 8.0, and (f) 9.0; Variations of *I*_pa_ of 0.010 M (**C**) matrine and (**D**) sophoridine at pH 9.0 and 4.0. (**E**) The oxidation peak current (*I*_pa_) of 0.010 M matrine in NaCl solutions containing esterase and urease with pH 6.5 (curve b) after subsequent 30 min reaction on adding 6 mM urea (curve a) and after 10 min reaction on adding 10 mM ethyl butyrate (EB) (curve c). (**F**) Dependence of *I*_pa_ on the alternate addition of EB and urea solutions for the same PDEA films.

The pH-sensitive property of PDEA film electrodes was demonstrated by the CV responses of matrine and sophoridine. From pH 9.0 to 4.0 at 25 °C, the *I*_pa_ of matrine and sophoridine decreased drastically with decrease of pH (Figure 2A,B). If the *I*_pa_ in NaCl solutions at pH 9.0 was set as the “on” state, whereas that at pH 4.0 as the “off” state, the CV response could be switched between the “on” and “off” state by switching the film electrode in the solutions between 9.0 and 4.0. This pH-dependent switchable CV behavior of the system could be repeated several times with good reversibility (Figure 2C,D). The response time of the on-off transitions was greatly influenced by the thickness of the PDEA hydrogel films. The average thickness of the films in water estimated by stereomicroscopy was 410 ± 30 μm at pH 9.0 and 25 °C. The response time increased with the thickness of PDEA hydrogel films (data not shown), implying that the diffusion of the drug molecules through the films to theelectrode surface for electron exchange became more difficult when the hydrogel films become thicker. In addition, oxidation peak currents of the drug molecules showed a linear relationship with the square root of scan rates from 0.05 to 1.0 V s^–1^ at pH 9.0 and 25 °C, suggesting diffusion-controlled behavior of the drug molecules.

The probable changes of structure and the average thickness of PDEA films at different pHs of solution were investigated by SEM (Figure S3) and stereomicroscopy (Table 1). The results showed that PDEA films have a similar network structure and surface morphology at pH 4.0 and 9.0 (Figure S3A,B). The PDEA component carries no charge regardless of the surrounding pH. In addition, the average thickness of the films at pH 9.0 (410 µm) estimated by stereomicroscopy was nearly the same as that at pH 4.0 (405 µm) (Table 1). In the control experiments, matrine and sophoridine demonstrated pH-sensitive CV properties at the bare GC electrode. When the pH increased from pH 4.0 to 9.0, the *I*_pa_ of matrine and sophoridine increased correspondingly (Figure S4A,B). Thus, the main mechanism was attributed to the property of matrine and sophoridine, which are alkaloid drugs with *pK*_a_ value of about 7.80−8.20 [31,32]. Their structure contains a tertiary amine nitrogen atom (^1^N), and an amide structure (^2^N) which has alkalinity. Amides cannot hydrolyze easily, so its main basicity is derived from ^1^N. The nitrogen atom in solution can provide lone pair electrons, and so the solutions become alkaline. When the pH decreased to 4.0, matrine was present in the form of a nitrogen cationic radical, which cannot transfer electrons to the electrode. As the amount of matrine in molecular form decreased, the oxidation peaks also decreased or even disappeared. When the pH increased to 9.0, matrine existed as a moleculer form, and the *I*_pa_ increased. In addition, the effect of the solution pH on the peak potentials was investigated (Figure S4). The oxidation peak potentials *E*_pa_ hardly shift with increasing pH 4.0–9.0, which means that the voltammetric behaviors of matrine and sophoridine are not a proton transfer process, just an electron transfer process between the drugs and the GC electrode under the experimental conditions.

**Figure 3:**
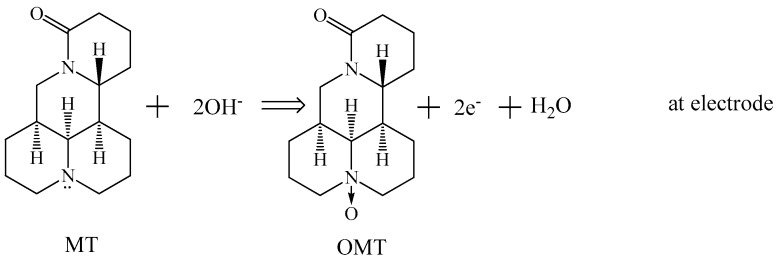
Oxidation mechanism of matrine (MT) on the GC electrode.

To verify the electron transfer mechanism between matrine and the electrodes, the electrochemical behavior of oxymatrine was further studied. The structures of oxymatrine and matrine are similar (Figure 3). No oxidation peak exists in the same potential window on the bare electrode in NaCl solutions at pH 9.0 (Figure S5), indicating that the electron transfer mechanism of matrine shown in Figure 3 is correct.

The pH of the solution can be controlled by in situ enzymatic catalysis [33,34,35]. The hydrolysis reaction of ethyl butyrate and urea was catalyzed by esterase (Est) and urease (Ur). For example, the film was immersed into the pH 6.5 NaCl solution containing esterase, urease, and matrine (Figure 2E, curve b). After the addition of ethyl butyrate, the pH of the solution began to decrease because of the production of butyric acid, and eventually reached 4.0. CV testing showed a small electrocatalytic oxidation peak (Figure 2E, curve c). Then urea was added, and the ammonia produced under urease catalysis caused an increase of pH. CV scanning was performed again, and the *I*_pa_ was markedly enhanced (Figure 2E, curve a). By adding ethyl butyrate and urea alternately to the enzyme solution of esterase and urease, the switchable behavior could be repeated many times between 4.0 and 9.0 (Figure 2F). 

### 3.3. Temperature-Responsive Behaviors of Poly(N,N-Diethylacrylamide) Films

**Figure 4:**
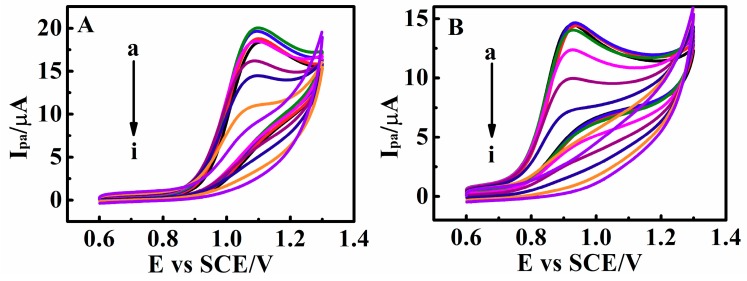

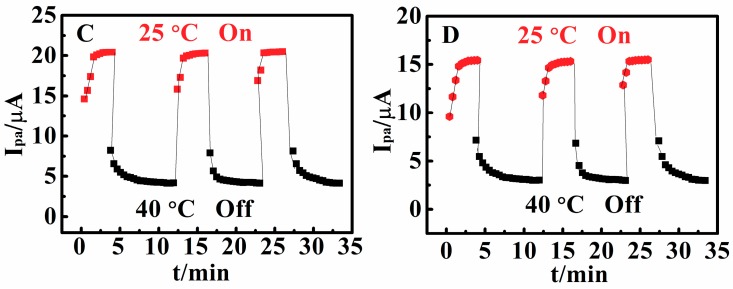
CVs of 0.010 M (**A**) matrine and (**B**) sophoridine for PDEA films at 0.05 V s^–1^ in NaCl solution with pH 9.0 containing no Na_2_SO_4_ at different temperatures: (a) 25 °C, (b) 27 °C, (c) 29 °C, (d) 31 °C, (e) 33 °C, (f) 35 °C, (g) 37 °C, (h) 39 °C, and (i) 40 °C. Variations of *I*_pa_ of 0.010 M (**C**) matrine and (**D**) sophoridine for PDEA films at 0.05 V s^–1^ in NaCl solutions with pH 9.0 at 25 °C and 40 °C.

The CV responses of matrine and sophoridine at the PDEA-film electrode were extremely sensitive to ambient temperature, with a lower critical phase transition temperature (LCST) of about 32 °C (Figure 4A,B), which was similar to that found in the literature [25,36]. When the solution temperature was below 32 °C, the *I*_pa_ values of matrine and sophoridine on PDEA-film electrode were extremely large in NaCl solutions at pH 9.0, but the CV peak current considerably decreased when the temperature exceeded 32 °C. The temperatures at 25 °C and 40 °C were selected to study the thermal-sensitive switching behavior of the film system: the film was at the “on” state at 25 °C and the “off” state at 40 °C. The temperature sensitive properties of PDEA hydrogel could be repeated many times, and the response time was about 3–6 min (Figure 4C,D). In the control experiments, the peak currents of matrine and sophoridine at the bare electrode showed no obvious change (Figure S6), and thus confirmed that the temperature sensitive property of this system mainly depends on the PDEA hydrogel films.

**Figure 5:**
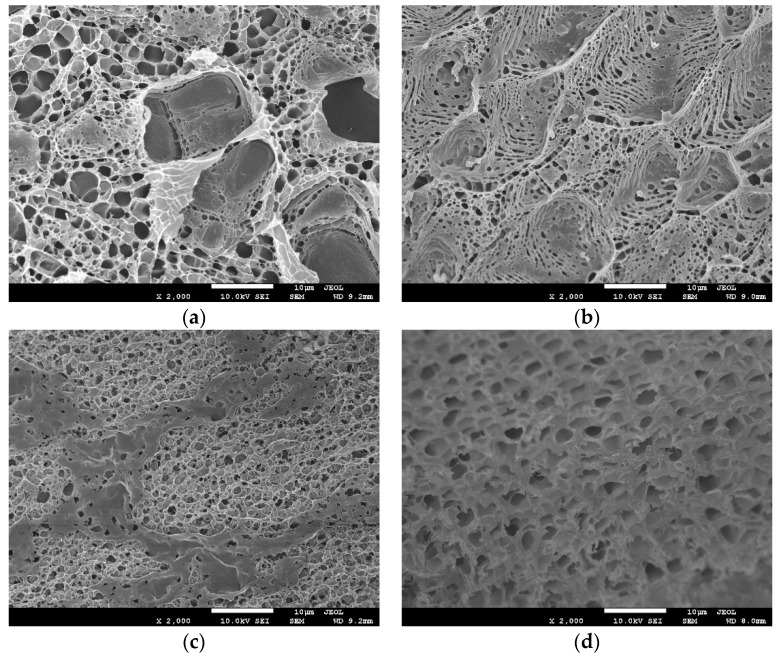
Top-view scanning electron micrographs (SEM) of PDEA hydrogel films on the GC electrode surface in NaCl solutions with pH 9.0 (**a**) containing no Na_2_SO_4_ and methanol at 25 °C, (**b**) containing 0.35 M Na_2_SO_4_ but no methanol at 25 °C, (**c**) containing no Na_2_SO_4_ and methanol at 40 °C, and (**d**) containing 20% methanol at 25 °C.

Scanning electron microscopy (SEM) also supported the structural changes of PDEA films with temperature. After being immersed in NaCl solutions with pH 9.0 at different temperatures, the films showed a network structure with numerous microsized pores and channels at 25 °C (Figure 5, panel a), whereas they displayed a compact and smooth surface at 40 °C (Figure 5, panel c). The structure change with temperature was further verified by the change of films thickness with temperature in water. The average film thickness estimated by stereomicroscopy at 40 °C became 276 µm, much smaller than that at 25 °C (410 µm) (Table 1).

### 3.4. Na_2_SO_4_-Responsive Behaviors of Poly(N,N-Diethylacrylamide) Films

**Figure 6:**
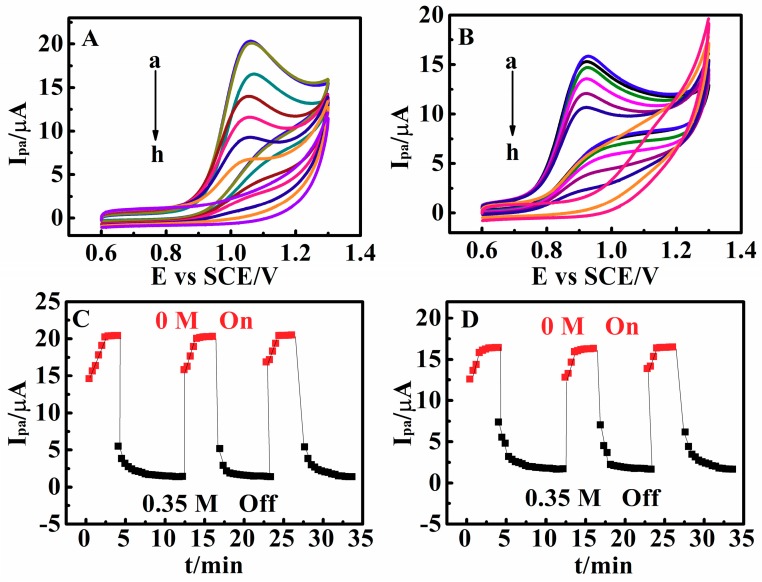
CVs of 0.010 M (**A**) matrine and (**B**) sophoridine for PDEA films at 0.05 V s^–11^ and 25 °C in NaCl solutions with pH 9.0 containing (a) 0 M, (b) 0.05 M, (c) 0.10 M, (d) 0.15 M, (e) 0.20 M, (f) 0.25 M, (g) 0.30 M and (h) 0.35 M Na_2_SO_4_. Variations of *I*_pa_ of (**C**) 0.010 M matrine and (**D**) 0.010 M sophoridine for PDEA films at 0.05 V s^–1^ and 25 °C in NaCl solutions with pH 9.0 containing 0 M and 0.35 M of Na_2_SO_4_.

The CV responses of matrine and sophoridine at PDEA-film electrodes were also sensitive to the salt concentration in solution. When the testing solution contained no Na_2_SO_4_ at 25 °C, matrine and sophoridine showed relatively large oxidation peaks, and the films were at the “on” state. When the solution contained 0.35 M Na_2_SO_4_ under the same conditions, the matrine and sophoridine oxidation peaks were undetectable, and the films were at the “off” state. The Na_2_SO_4_-sensitive switching behaviors of matrine and sophoridine were also reversible. The “on” and “off” states of the system could be repeated several times (Figure 6C,D). For the control experiments, the peak currents of the drug probes at the bare electrode showed no distinct change in solutions containing 0 and 0.35 M Na_2_SO_4_ (Figure S7). That is, the Na_2_SO_4_-sensitive CV behaviors of matrine and sophoridine also should be ascribed to the PDEA component in the films.

Other types of salts, such as MgSO_4_, NaNO_3_, and NaCl, can also cause the phase transition of PDEA film and the CV switchable behavior of drug probes (Figure S8). The main difference between these salts was their different critical phase change concentration (SO_4_^2^^−^ (0.20 M) < Cl^−^ (0.54 M) < NO_3_^−^ (0.95 M). Na_2_SO_4_, NaCl, and NaNO_3_ contain the same cationic Na^+^ but different anions. The result is consistent with previous work [28]. The main difference is that this salt sensitivity can be used as an input signal to construct a logic gate system of drug molecules. According to the above test results, we selected Na_2_SO_4_ as the representative salt to discuss the sensitivity of the PDEA films. Na_2_SO_4_ caused the phase transition of the PDEA film, which was essentially the same as the temperature-induced phase change [37,38]. When the Na_2_SO_4_ concentration was lower than the critical concentration, PDEA exhibited a loose linear structure in which drugs easily diffused, resulting in increased CV response. When the Na_2_SO_4_ concentration became higher than its critical concentration, the hydrogen bonds between PDEA and water molecules considerably weakened, leading to a dense globular structure and preventing the drug molecules from reaching the electrode surface for electron exchange. The structural changes caused by the salt concentration can be further demonstrated by SEM (Figure 5, panel a and b) and stereomicroscopy (Table 1). The average thickness of PDEA films was 207 µm in the presence of 0.35 M Na_2_SO_4_, much smaller than 410 µm in the absence of Na_2_SO_4_ (Table 1).

### 3.5. Methanol-Responsive Behaviors of Poly(N,N-Diethylacrylamide) Films

**Figure 7:**
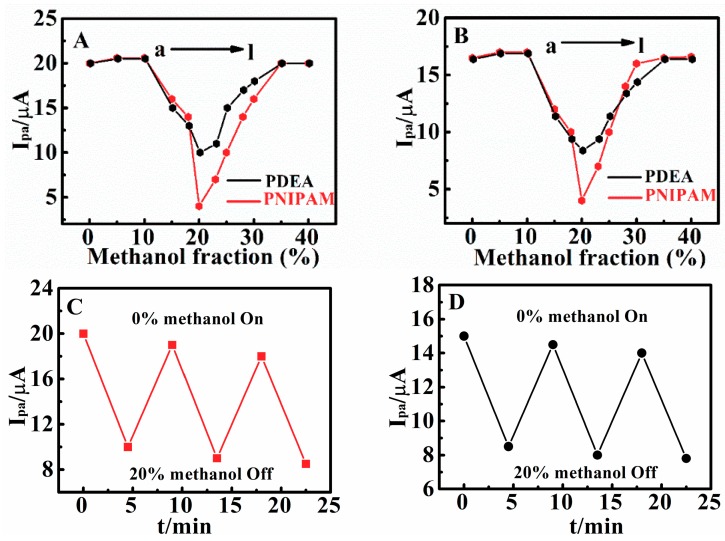
Influence of methanol volume fraction in water/methanol mixture solvent on CV *I*_pa_ of 0.010 M (**A**) matrine and (**B**) sophoridine at 0.05 V s^−1^ in pH 9.0 solutions for PDEA and poly (*N*-isopropylacrylamide) PNIPAM films at 25 °C. Variations of *I*_pa_ of (**C**) 0.010 M matrine and (**D**) sophoridine for PDEA films at 0.05 V s^−1^ and 25 °C in NaCl solutions with pH 9.0 containing 0% and 20% methanol.

The CV response of matrine and sophoridine on the PDEA electrode is also sensitive to the fraction of methanol in the mixture of water. When the content of methanol was lower than 20%, the CV responses of the drug molecular probes were very large and the peak currents were basically the same, indicating that the methanol content had little effect on the CV response. However, the CV peak current decreased to the minimum value at 10 µA when the content of methanol was between 20% and 30%. The behavior is reversible and can be repeated over several cycles (Figure 7C,D). When the content of methanol continues to increase, the CV response signals of the drug probes will increase with the increasing methanol content (Figure 7A,B). The switchable behavior is reversible and can be repeated over several cycles (Figure 7C,D), and only a very small decrease of *I*_pa_ is observed with the increase of cycles, although the reason for the decrease is not yet clear.

In the control experiments, the oxidation peak currents of matrine and sophoridine at the bare electrode showed no significant difference between the solution containing 0% and 20% methanol (Figure S9). We assumed that this is related to the structure change of PDEA. In previous literature, it was reported [39,40,41] that due to the inability of the tertiary amide groups in PDEA structures to form intramolecular hydrogen bonds, a compact spherical structure could not be formed, unlike PNIPAM which has a secondary amide group, which can form both intramolecular hydrogen bonds and extramolecular hydrogen bonds. Therefore, PNIPAM has a coil-to-globule-to-coil transition in water/methanol solutions of different proportions. This was further demonstrated by electrochemical signal testing (Figure 7). When the methanol fraction reaches 20%, the oxidation peak current heights of matrine and sophoridine decrease to 4.2 µA and 4.1 µA on the PNIPAM film electrode, respectively. However, their oxidation peak current heights merely reach 10 µA and 8.5 µA on PDEA film electrode, respectively. Meanwhile, we compared PDEA surface with PNIPAM using SEM (Figure S10). We found that the pore diameter of PNIPAM was much smaller than PDEA after 20% methanol treatment, which further supported the view that PDEA cannot form a compact spherical structure. The structural changes caused by the methanol fraction could be further demonstrated by stereomicroscopy (Table 1). The average thickness of PDEA films was 328 µm in the presence of 20% methanol. The result proves that PDEA hydrogel was not fully contracted (Table 1).

### 3.6. Electrocatalysis by Carboxylated Multi-Walled Carbon Nanotubes

In the logic gate system, adding an input is relatively easy, but adding an output signal is difficult. Even so, people are still working in this direction, because the logic gate with more output has a faster processing speed and stronger processing capacity, which can solve more complex practical problems. To increase the complexity of the logic gate circuit in this experiment, we used electrocatalytic amplification to increase the oxidation peak current of the drug probes and the number of threshold values.

Due to their unique properties and potential applications, carbon nanotubes (CNTs) have become a hot topic worldwide. CNTs are divided into MWCNTs and single-walled carbon nanotubes (SWCNTs). MWCNTs consist of cylindrical graphene sheets of nanometer-scale diameters [42,43,44], which show unique properties, such as high electrical conductivity [45,46,47], high chemical stability [48,49], and extremely high mechanical strength and modulus [44,46]. However, their application is limited by the poor dispersion of MWCNTs. Hence, we prepared carboxylated multiwalled carbon nanotubes (c-MWCNTs) with good dispersion. The preparation methods and characterization are described in Supplementary materials. c-MWCNTs are often constructed on the electrode surface and used as electrochemical sensors to determine compounds. For example, Heidarimoghadam’s group [50] developed a novel method for quantifying furosemide in biological fluids based on the electro-reduction of Zn(II)–furosemide complex at a GC electrode modified with c-MWCNTs. The sensor was used for quantifying furosemide in drug and biological fluid samples. Bhawna and coworkers [51] explored an improved amperometric glutamate biosensor fabricated by immobilization of glutamate oxidase onto a hybrid of c-MWCNTs and gold nanoparticles attached onto a gold electrode through chitosan film, which showed remarkably enhanced sensitivity and selectivity for glutamate.

**Figure 8:**
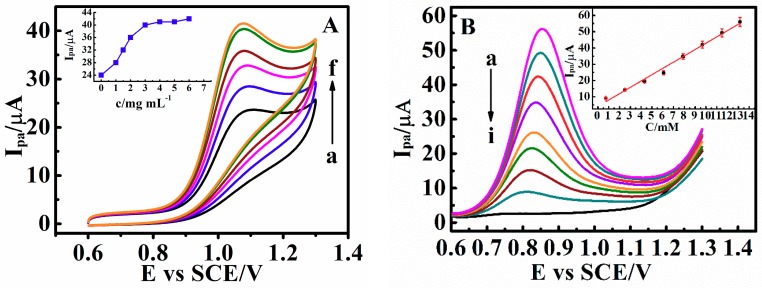
(**A**) CV catalytic oxidation peak currents (*I*_pa_) of 0.010 M matrine at 0.05 V s^−1^ and 25 °C for PDEA films in NaCl solutions with pH 9.0 containing (a) 0, (b) 1, (c) 2, (d) 3, (e) 4 and (f) 5 mg mL^−1^ of c-MWCNTs. (Inset: The relationship between *I*_pa_ and concentration of c-MWCNTs). (**B**) Differential pulse voltammetry (DPV) response of c-MWCNTs/PDEA on a GC electrode to the additions of matrine concentration from (a) 0 mM to (i) 13.1 mM (Each incremental concentration is 1.5 mM) in NaCl solutions with pH 9.0 (Inset: The relationship between the *I*_pa_ and matrine concentration).

In our work, we constructed c-MWCNTs as the catalytic layer and PDEA as the outside stimulus response layer. Electrocatalytic amplification enables higher oxidation peak currents, which can increase the sensor’s threshold and the complexity of the logic gate. Through continuous testing, the optimum concentration of c-MWCNTs in combination with drug probes was 5 mg mL^−1^ (Figure 8A). The *I*_pa_ of matrine at c-MWCNTs/PDEA-film electrode increased considerably in comparison with the PDEA-film electrode (Figure 8A, curves a and f). The effective area of the electrode surface can be increased because of the large specific surface area of c-MWCNTs. The electron transfer between the electroactive molecules and electrode can be greatly promoted by c-MWCNTs due to its excellent conductivity. In addition, the coverage of c-MWCNTs on the electrode surface can also improve the porosity of the films. The linear range of the calibration curve for matrine is from 1.50 to 13.10 mM (Figure 8B, Inset) and could be expressed by the equation *I*_(MT)_ (µA) = 2.895 + 4.040·*c*_(MT)_ (mM) (R = 0.995), the detection limit is 2.01 × 10^−4^ M (S/N = 3). Similarly, the oxidation peak current of sophoridine is also proportional to its concentration range of 5.40–18.70 mM in solution, and the linear equation is *I*_(SR)_ (µA) = 3.642 + 3.681·*c*_(SR)_ (mM), (R = 0.996) and the detection limit is 1.98 × 10^−4^ M (S/N = 3). To examine the repeatability of the system, five c-MWCNTs/PDEA film electrodes were fabricated independently under the same conditions, and each of the five sensors was used to detect 10.00 mM matrine and sophoridine by repeated elution and determination in triplicate. The relative standard deviations (RSD) are 4.5% and 4.8%, respectively, suggesting that the system has good repeatability.

### 3.7. Switchable Electrocatalysis Controlled by pH, Temperature, SO_4_^2−^ Concentration and Methanol

**Figure 9:**
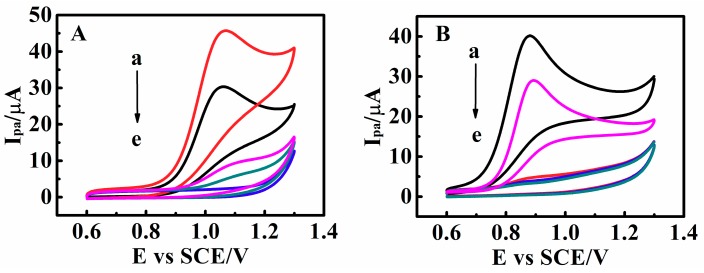
CV catalytic oxidation peak currents (*I*_pa_) of 0.010 M (**A**) matrine and (**B**) sophoridine at 0.05 V s^−1^ for c-MWCNTs/PDEA films (a) in NaCl solution with pH 9.0 at 25 °C in the absence of Na_2_SO_4_ and methanol, (b) in NaCl solution with pH 9.0 at 25 °C containing 20% methanol but no Na_2_SO_4_, (c) in NaCl solution with pH 9.0 at 25 °C containing 0.35 M Na_2_SO_4_ but no methanol, (d) in NaCl solution with pH 9.0 at 40 °C in the absence of Na_2_SO_4_ and methanol, and (e) in NaCl solution with pH 4.0 at 25 °C containing no Na_2_SO_4_ and methanol.

Matrine and sophoridine exhibited pH-, temperature-, SO_4_^2−^- and methanol-sensitive CV behavior on the c-MWCNTs/PDEA electrodes (Figure 9). For example, at pH 9.0 and 25 °C, when the c-MWCNTs/PDEA-film electrode was placed in the solution containing matrine without SO_4_^2−^ and methanol, a large CV catalytic oxidation wave was observed (Figure 9A, curve a). However, when the same c-MWCNTs/PDEA film was placed in the same solution at 40 °C, the electrocatalytic response became extremely small (Figure 9A, curve c) because the film was turned “off” for matrine at 40 °C, leading to the disruption of catalytic reactions. Therefore, the electrocatalytic oxidation of matrine can be switched “on–off” states by regulating the temperature of the solution between 25 °C and 40 °C. This temperature-sensitive and controllable electrocatalysis is reversible and can be repeated many times (Figure S11B). In addition, the CV oxidation peak current ratio, *I*_pa25_/*I*_pa40_, could be amplified by electrocatalysis, where *I*_pa25_ and *I*_pa40_ represented CV oxidation peak currents at 25 °C and 40 °C for the same c-MWCNTs/PDEA films, respectively. At the PDEA-film electrode in NaCl supporting solutions containing 0.010 M matrine, the *I*_pa25_/*I*_pa40_ ratio was about 8.3, whereas the ratio increased to about 15 at the c-MWCNTs/PDEA-film electrode. The electrocatalysis of matrine for the system can be modulated by SO_4_^2−^ concentration. This electrocatalytic behavior was at the “on” state in NaCl solutions containing matrine without SO_4_^2−^ at 25 °C (Figure 9A, curve a). By contrast, the system was turned “off” when the SO_4_^2−^ concentration was 0.35 M (Figure 9A, curve b). SO_4_^2−^-triggered electrocatalysis of matrine can also be switched between the “on” and “off” states repeatedly and reversibly (Figure S11C). The electrocatalysis of matrine can be modulated by pH, but the regulation mechanism is different from the temperature and SO_4_^2−^. Temperature and SO_4_^2−^ primarily affected the PDEA part of the binary c-MWCNTs/PDEA structure, whereas pH primarily affected the existing forms of the drug probes, causing them to exist as either molecules or ions. For example, matrine exists as a molecule at pH 9.0; when the c-MWCNTs/PDEA-film electrode was placed in the solution containing 0.010 M matrine without SO_4_^2−^ at 25 °C, a large CV catalytic oxidation wave appeared (Figure 9A, curve a). However, at pH 4.0, the electrocatalytic response became extremely small (Figure 9A, curve e). pH-triggered electrocatalysis of matrine could also be switched between the “on” and “off” states repeatedly and reversibly (Figure S11A). The effect of methanol is the most special. The presence of methanol can actually make the *I*_pa_ height of the drug probes change at the PDEA film electrode, however, when the fraction of methanol is 25%, the peak current cannot reach the minimum point, that is, the PDEA film cannot be completely closed. In other words, when the solution contains no methanol, matrine showed a large oxidation peak current, however, when the methanol proportion increased to 20%, the peak currents decreased to 30 µA and 28 µA, respectively (Figure 9A,B, curve b), and this behavior could be repeated over many cycles (Figure S11D). The temperature-, salt- and methanol- sensitive behavior of the film electrodes mainly depends on the morphological changes and swelling, which have good reproducibility and were demonstrated by SEM and stereomicroscopy results (Figure 5 and Table 1). However, the structure change of the films with pH is less possible for our system.

### 3.8. Establishment of a 6-Input/5-Output Logic Gate

**Figure 10:**
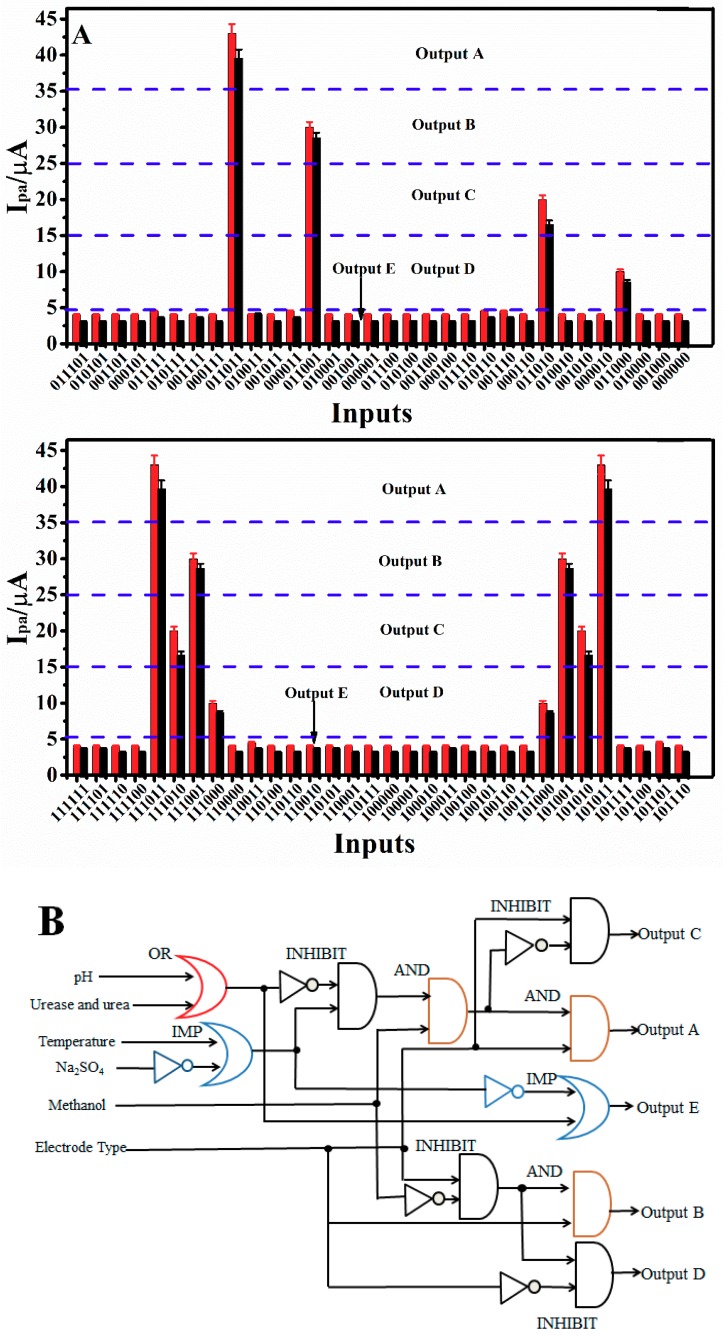
(**A**) Catalytic oxidation peak currents (*I*_pa_) of 0.010 M matrine and sophoridine for the c-MWCNTs/PDEA films at 0.05 V s^−1^ in the solution as the output with all possible 64 combinations of 6 inputs. The threshold is marked by dashed lines. (**B**) The symbolic representation of the logic gate circuit.

The foundation of advanced and more complicated molecular logic devices is composed of basic or simple binary logic gates. A 6-input/5-output logic gate was developed on the basis of the above experimental results. The definitions of the six inputs are described in Table 2. The *I*_pa_ absolute values of matrine and sophoridine at the c-MWCNTs/PDEA-film electrodes within five different ranges were divided into five outputs with four thresholds (35, 25, 15, and 5 μA) according to different mechanisms. The 64 possible combinations of the six inputs and corresponding outputs are listed in Table S1, and the corresponding diagram is presented in Figure 10A. The Output A (*I*_pa_ ≥ 35 µA), of which input combinations were (011011), (111011), and (101011), was at the “1” state, and Outputs B, C, D, and E were at the “0” state. In these three cases, the films not only assumed a swollen structure but also the inner layer of the GC electrode contained c-MWCNTs. The electrocatalysis of drug probes by c-MWCNTs thus resulted in the largest CV response. The Output B (35 µA < *I*_pa_ ≤ 25 µA), which input combinations were (101001), (011001)and (111001), was at the “1” state, and Outputs A, C, D, and E were at the “0” state. When the methanol content was 20%, PDEA was not completely closed. c-MWCNTs would catalyze matrine and sophoridine to increase the oxidation peak current. With input combinations of (011010), (111010), and (101010), Output C (25 µA < *I*_pa_ ≤ 15 µA) was at the “1” state, while Outputs A, B, D, and E were at the “0” state. In these three cases, the PDEA films also assumed a swollen structure, but the inner layer of the GC electrode did not contain c-MWCNTs. Electrocatalysis is almost impossible in this situation, but the drugs were still able to diffuse through the films and easily transfer electrons to the electrode, thus resulting in a mid-range CV response. With input combinations of (011000), (111000), or (101000), Output D (15 µA < *I*_pa_ ≤ 5 µA) was at the “1” state, while Outputs A, B, C, and E were at the “0” state. When the solution contains 20% methanol and there is no c-MWCNTs layer assembled in the film electrode, PDEA hydrogel films cannot completely close and drug molecules can partially penetrate to the electrode surface and generate electrochemical signals. Output E (*I*_pa_ < 5 µA) was at “1” state in the remaining 52 combinations, while Outputs A, B, C, and D were at the “0” state. Under these conditions, the PDEA films changed into a compact structure, the drug molecular probes hardly diffused through the films to reach the electrode surface, or the drugs were present in the form of a nitrogen cationic radical, or both of these things happened at the same time, leading to an extremely small CV signal. The response characteristics and thresholds of each output are summarized in Table 3. The symbolic representation of the 6-input/5-output logic gate is visualized in Figure 10B, which shows the combination of OR, NOT, INHIBIT, AND, and IMP (IMPLICATION) gates [2,10,52].

### 3.9. Fabrication of a 2-to-1 Encoder

**Figure 11:**
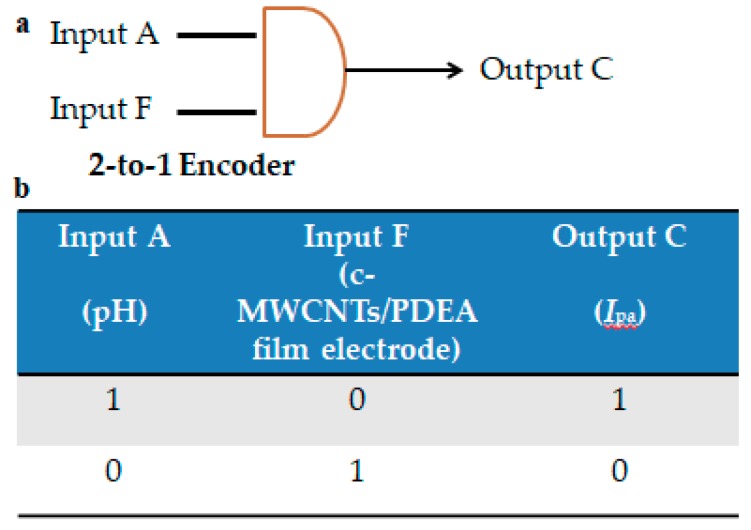
(**a**) Schematic representations and (**b**) truth tables of 2-to-1 encoder.

An encoder is a logic device that can compress information. Thus, the function of the 2-to-1 encoder is to compress 2 inputs into 1 output [53]. Herein, to establish the 2-to-1 encoder, pH (Input A) and c-MWCNTs/PDEA binary electrode (Input F) were chosen as the two inputs, and one of the CV peak currents at 1.02 V or 0.91 V (Output C) was selected as the single output in the 0.010 M drug probe solution with no SO_4_^2^^−^ and methanol at 25 °C. Output C in the above logic gate system was selected as the sole output, so that these definitions were consistent with those in the above logic gate system. For this device, if the input was set at (1,0), Output C would be at the “1” state at PDEA film electrodes in NaCl solutions at pH 9.0, the presence of the drug in the pH 9.0 solution is molecular, which would lead to a CV response larger than the threshold of 15 µA. If the input changed to (0,1), however, Output C would be at the “0” state because neither matrine nor sophoridine can exist in molecular form. The truth table of this simple 2-to-1 encoder is presented in Figure 11 accompanied with the schematic representation. It should be understood that for all of the non-explicitly defined input combinations such as (0,0) and (1,1), the corresponding outputs are treated as irrelevant [53].

## 4. Conclusions

The multi-responsive c-MWCNTs/PDEA films were successfully prepared on GC electrodes through combination of c-MWCNT inner layers and PDEA hydrogel surface layers. The films exhibited reversible pH-, temperature-, SO_4_^2−^ and methanol dependent CV on–off behavior toward the drug molecules matrine and sophoridine. This multi-triggered switchable behavior can be used to achieve the multiple controllable electrochemical oxidation of matrine and sophoridine catalyzed by c-MWCNTs. Although numerous studies have reported on multi-signal controlled electrochemistry [54,55,56], this unique system includes the following points: (1) The combination of the c-MWCNTs/PDEA dual structure film and drug molecular eletrocatalysis was used to construct a logic gate network and device for the first time; (2) Works on multi-switchable electrocatalysis of drugs, especially those used in traditional Chinese medicine, have been extremely limited to date; (3) The complexity of the logical gate system was increased by increasing the input of the stimulus, which was divided into five different CV oxidation peaks ranges according to different mechanisms; (4) The methanol sensitive property of PDEA hydrogel films was investigated by the CV method. Because the PDEA hydrogel films could not be closed completely upon external stimuli of methanol, the drug molecules still could be catalyzed by the c-MWCNTs inner layer. This work presents several potential applications and some bold predictions. Although the current work is still in the preliminary research stage, it does reflect the progress of a logic gate network. The multi-sensitive interface was coupled with electrocatalysis so that the logic gate system could be developed, thereby providing a novel approach to solve certain problems, such as biomedical diagnosis, the design of novel drug sensors, and molecular devices in the future.

## Figures and Tables

**Table 1:** Average thickness of poly(*N*,*N*-diethylacrylamide (PDEA) films estimated by stereomicroscopy under different conditions.

Measurement Conditions	Average Thickness/µm
pH 9.0 and 25 °C	410 ± 30
pH 4.0 and 25 °C	405 ± 8
pH 9.0 and 40 °C	276 ± 15
pH 9.0 and 25 °C containing 0.35 M Na_2_SO_4_	207 ± 20
pH 9.0 and 25 °C containing 20% methanol	328 ± 18

**Table 2:** Definition of the six inputs for the system.

Input	Definition	The “1” State	The “0” State
**A**	pH	9.0	5.0
**B**	Urease and urea	6 mM urea	0 mM urea
**C**	Temperature	25 °C	40 °C
**D**	Na_2_SO_4_	0.35 M	0 M
**E**	Methanol	0%	20%
**F**	Electrode Type	c-MWCNTs/PDEA	PDEA

**Table 3:** A summary of the characteristics and thresholds of the output signals.

Outputs	PDEA States	Drug States	Methanol Fraction	Electrode Type
**Output A** **(*I*_pa_ ≥ 35 µA)**	No phase transition	molecule	0%	c-MWCNTs/PDEA electrode
**Output B** **(35 µA < *I*_pa_ ≤ 25 µA)**	No phase transition	molecule	20%	c-MWCNTs/PDEA electrode
**Output C** **(25 µA < *I*_pa_ ≤ 15 µA)**	No phase transition	molecule	0%	PDEA electrode
**Output D** **(15 µA < *I*_pa_ ≤ 5 µA)**	No phase transition	molecule	20%	PDEA electrode
**Output E** **(*I*_pa_ < 5 µA)**	The phase transition of PDEA was appeared or drugs were present in the form of nitrogen cationic radical or both of these things happen at the same time			

## Supplementary Materials

The following are available online at http://www.mdpi.com/1424-8220/18/10/3358/s1, Figure S1. Fourier-transform infrared spectrum (FTIR) of (**a**) *N*,*N*-diethylacrylamide (DEA) and (**b**) poly(*N*,*N*-diethylacrylamide) (PDEA). Figure S2. Chemical structure of (**a**) matrine (MT) and (**b**) sophoridine (SR) on GC electrode. Figure S3. Top-view SEM of PDEA films on glassy carbon electrode surface in NaCl solutions with (**A**) pH 4.0 and (**B**) pH 9.0. Figure S4. CVs of *I*_pa_ for 0.01 M (**A**) matrine and (**B**) sophoridine at 0.05 V s^−1^ for bare GC electrode in pH (**a**) 4.0, (**b**) 5.0, (**c**) 6.0, (**d**) 7.0, (**e**) 8.0 and (**f**) 9.0 NaCl solutions. Effects of different pH on the oxidation peak current and oxidation peak potential of (**C**) matrine and (**D**) sophoridine. Figure S5. CVs of *I*_pa_ for 0.01 M (**a**) matrine and (**b**) oxymatrine at 0.05 V s^−1^ in pH 9.0 NaCl solutions at bare GC electrode. Figure S6. CVs of *I*_pa_ for 0.01 M (**A**) matrine and (**B**) sophoridine at 0.05 V s^−1^ and 25 °C in pH 9.0 NaCl solutions at (**a**) 25 and (**b**) 40 C on bare GC electrodes. Figure S7. CVs of *I*_pa_ for 0.01 M (**A**) matrine and (**B**) sophoridine at 0.05 V s^−1^ and 25 °C in pH 9.0 NaCl solutions containing (**a**) 0 and (**b**) 0.35 M on bare GC electrodes. Figure S8. Dependence of the catalytic oxidation peak current (*I*_pa_) of 0.01 M sophoridine at 0.05 V s^−1^ and 25 °C for PDEA films in NaCl solutions with pH 9.0 containing (**a**) Na_2_SO_4_, (**b**) MgSO_4_, (**c**) NaCl, and (**d**) NaNO_3_. Figure S9. CVs of *I*_pa_ for 0.01 M (**A**) matrine and (**B**) sophoridine at 0.05 V s^−1^ and 25 °C in pH 9.0 NaCl solutions containing (**a**) 0% and (**b**) 20% methanol on bare GC electrodes. Figure S10. Top-view scanning electron micrographs SEM of (**a**) PDEA and (**b**) PNIPAM hydrogel films on GC electrode surface in NaCl solutions containing 20% methanol. Figure S11. Dependence of cyclic voltammetry catalytic oxidation peak currents (*I*_pa_) for c-MWCNTs/PDEA films at 0.05 V s^−1^ in solutions containing 0.01 M matrine when the system was switched between (**A**) pH 4.0 and 9.0 at 25 °C, (**B**) 25 and 40 °C at pH 9.0, (**C**) 0 M and 0.35 M Na_2_SO_4_ at pH 9.0 and at 25 °C and (**D**) 0% and 20% methanol at pH 9.0 and at 25 ºC. Figure S12. Fourier-transform infrared spectrum (FTIR) of (**a**) c-MWCNTs and (**b**) MWCNTs samples. Figure S13. Transmission electron microscopy (TEM) of (**a**) MWCNTs and (**b**) c-MWCNTs. Table S1. Truth table.
