# Supplementary material for: Construction of Multiple Switchable Sensors and Logic Gates Based on Carboxylated Multi-Walled Carbon Nanotubes/Poly(N,N-Diethylacrylamide)
Source: Sensors (Basel). 2018 Oct 8;18(10):3358. doi: 10.3390/s18103358 (PMC6211007; doi:10.3390/s18103358)
Supplement: Supplementary file 1 [file sensors-18-03358-s001.pdf]

Supplementary Materials

Construction of multiple switchable sensors and logic gates based on carboxylated multi-walled carbon nanotubes/Poly (N,N-diethylacrylamide)

Xuemei Wu[1],† Xiaoqing Bai[2],† Yang Ma[1],† Jie Wei[1], Juan Peng[3], Keren Shi[4], Huiqin Yao[2]*

[1] College of Pharmacy, Ningxia Medical University, Yinchuan 750004, P. R. China, Xuemei_WU157@163.com
[2] School of Basic Medical Sciences, Ningxia Medical University, Yinchuan 750004, P. R. China
[3] School of Chemistry and Chemical Engineering, Ningxia University, Yinchuan 750021, P. R. China, pengjuan@nxu.edu.cn
[4] State Key Laboratory of High-efficiency Coal Utilization and Green Chemical Engineering, Ningxia University, Yinchuan 750021, P. R. China, shikeren@163.com

*Correspondence: huiqin_yao@163.com Tel: (86)-951-698-0110

†These authors contributed equally to this work.

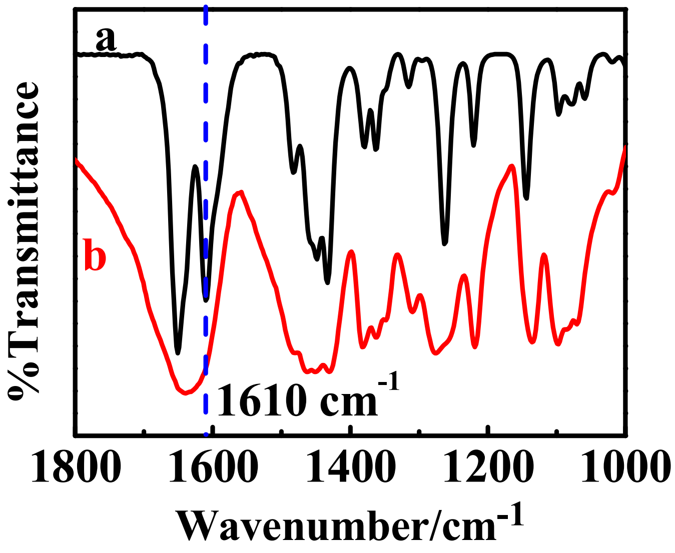

Figure S1. Fourier-transform infrared spectrum (FTIR) of (a) N,N-diethylacrylamide (DEA) and (b) poly(N,N-diethylacrylamide) (PDEA).

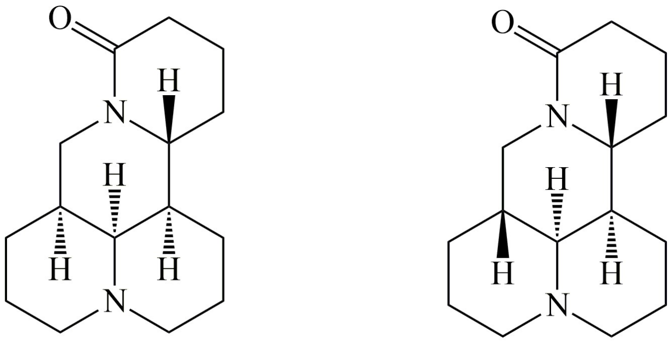

(a) **(b)**

Figure S2. Chemical structure of (a) matrine (MT) and (b) sophoridine (SR) on GC electrode.

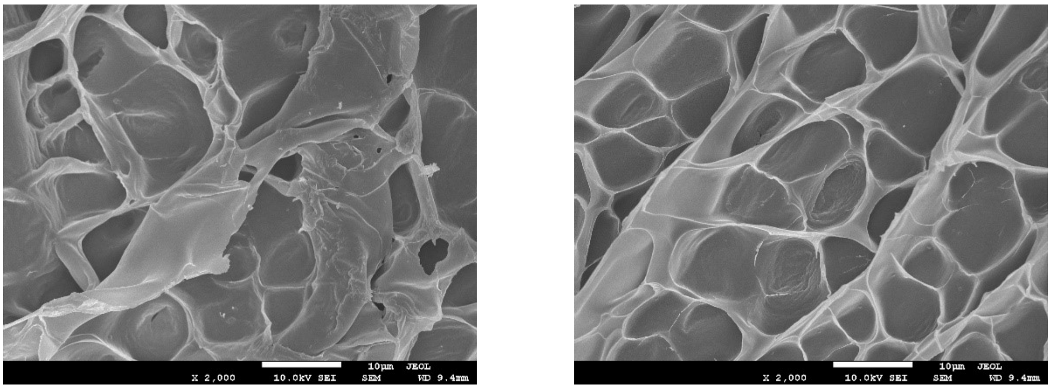

(a) (b)

Figure S3. Top-view SEM of PDEA films on GC electrode surface in NaCl solutions with (A) pH 4.0 and (B) pH 9.0.

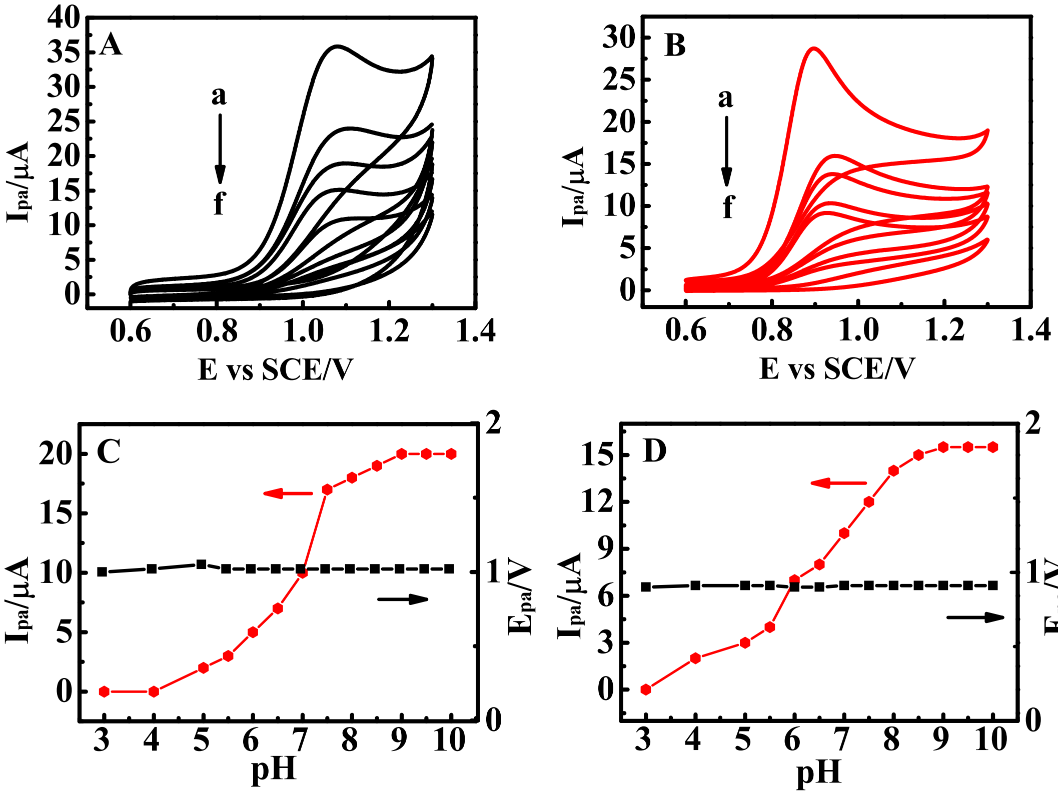

Figure S4 CVs of I_{pa} for 0.010 M (A) matrine and (B) sophoridine at 0.05 V s^{-1} for bare GC electrode in pH (a) 4.0, (b) 5.0, (c) 6.0, (d) 7.0, (e) 8.0 and (f) 9.0 NaCl solutions. Effects of different pH on the I_{pa} and oxidation peak potential (E_{pa}) of (C) matrine and (D) sophoridine.

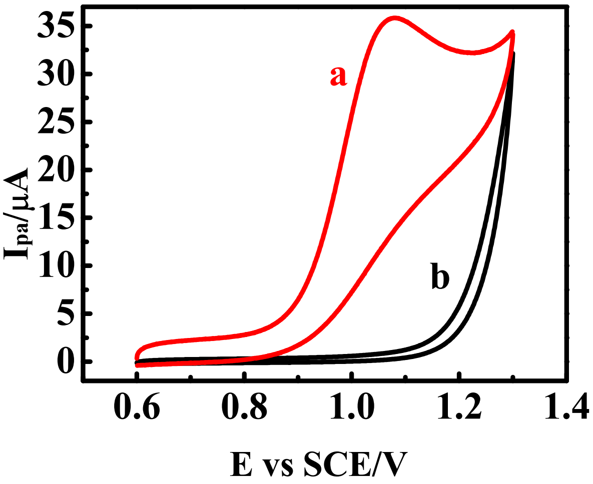

Figure S5 CVs of I_{pa} for 0.010 M (a) matrine and (b) oxymatrine at 0.05 V s^{-1} in pH 9.0 NaCl solutions at bare GC electrode.

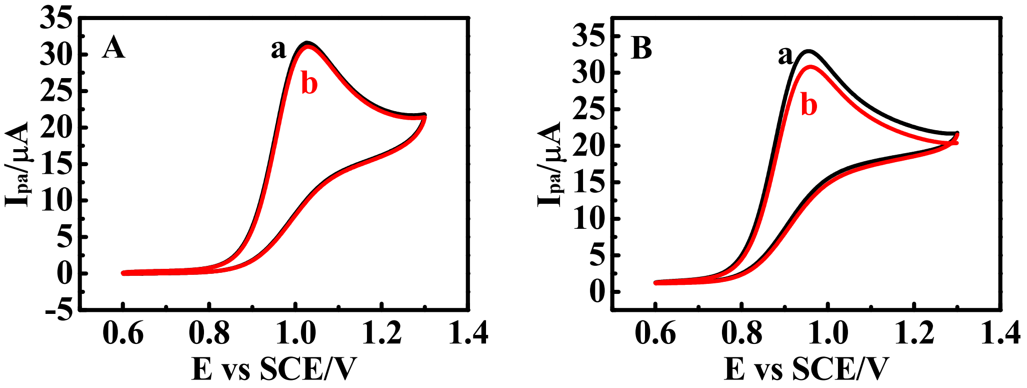

Figure S6 CVs of I_{pa} for 0.010 M (A) matrine and (B) sophoridine at 0.05 V s^{-1} and 25 °C in pH 9.0 NaCl solutions at (a) 25 and (b) 40 °C at bare GC electrodes.

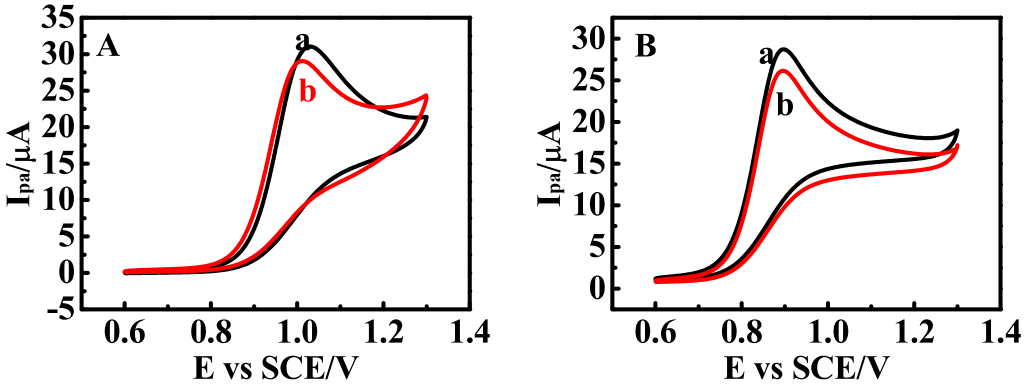

Figure S7 CVs of I_{pa} for 0.010 M (A) matrine and (B) sophoridine at 0.05 V $^{-1}$ and 25 °C in pH 9.0 NaCl solutions containing (a) 0 and (b) 0.35 M at bare GC electrodes.

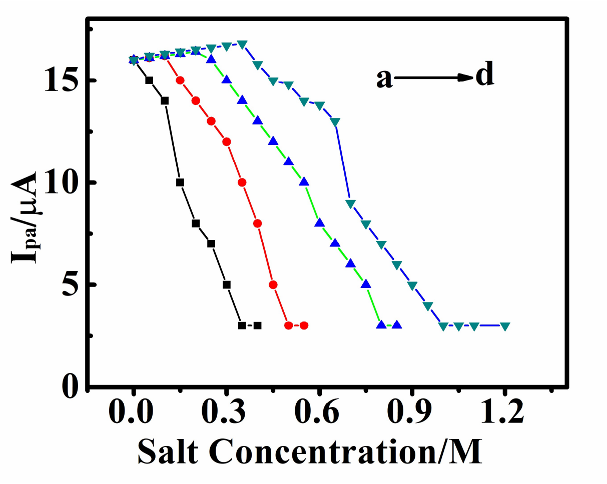

Figure S8. Dependence of the catalytic oxidation peak current (I_{pa}) of 0.010 M sophoridine at 0.05 V s^{-1} and 25 °C for PDEA films in NaCl solutions with pH 9.0 containing (a) Na_2SO_4, (b) $MgSO_4$, (c) NaCl, and (d) $NaNO_3$.

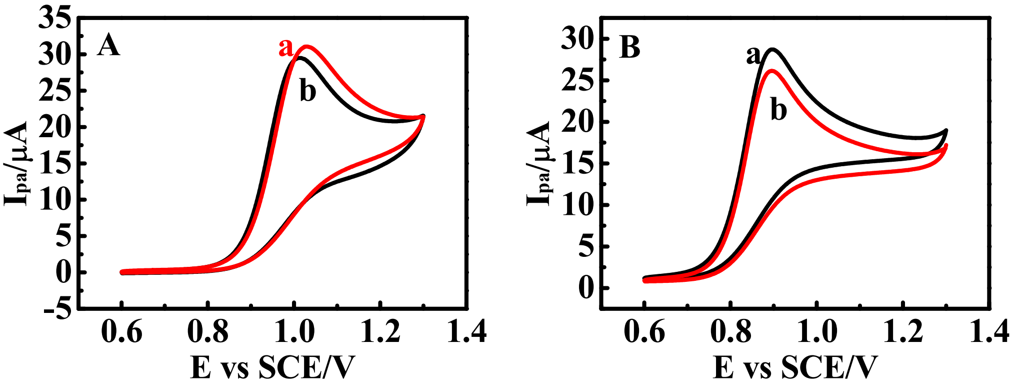

Figure S9 CVs of I_{pa} for 0.010 M (A) matrine and (B) sophoridine at 0.05 V s^{-1} and 25 °C in pH 9.0 NaCl solutions containing (a) 0% and (b) 20% methanol at bare GC electrodes.

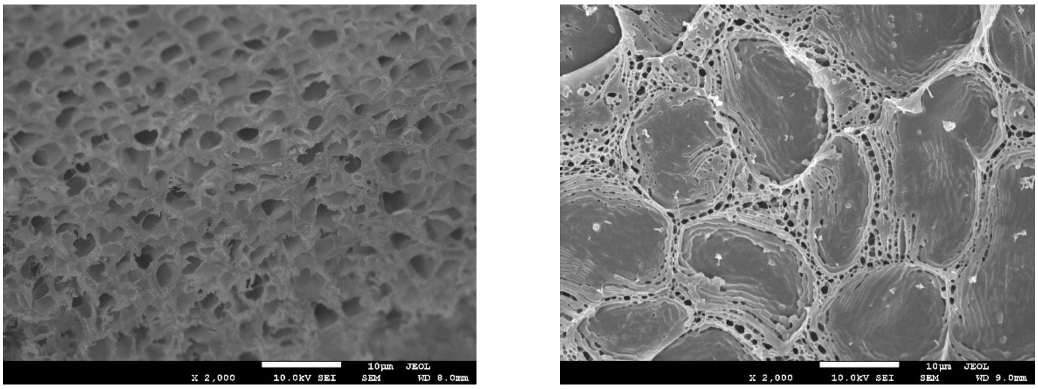

(a) **(b)**

Figure S10 Top-view SEM of (a) PDEA and (b) PNIPAM hydrogel films on GC electrode surface in NaCl solutions containing 20% methanol.

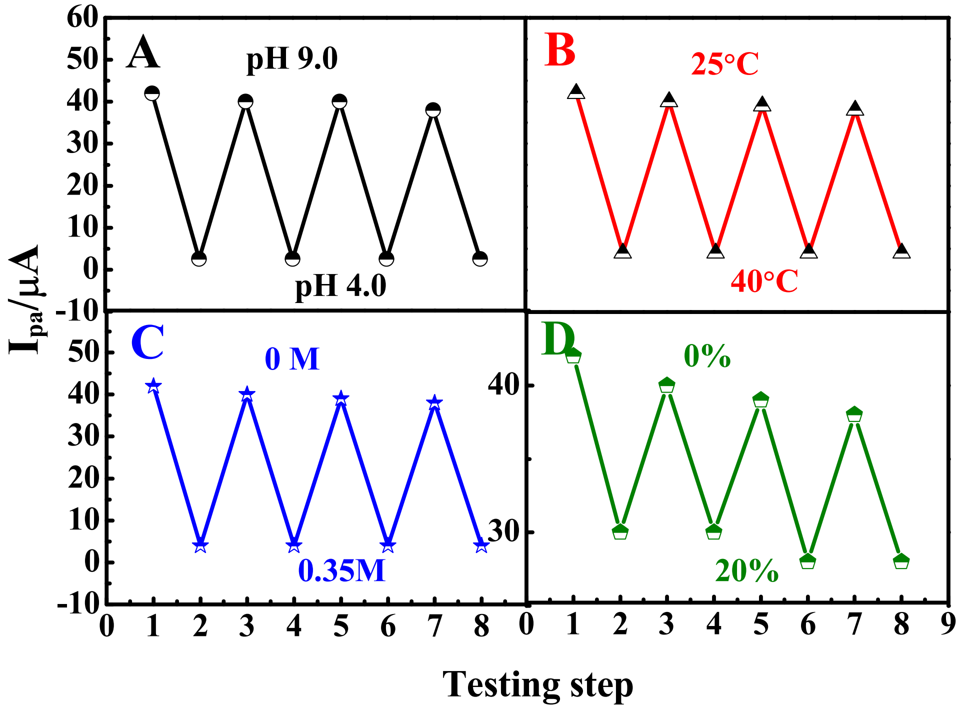

Figure S11. Dependence of cyclic voltammetry catalytic oxidation peak currents (I_{pa}) for c-MWCNTs/PDEA films at 0.05 V s^{-1} in solutions containing 0.010 M matrine when the system was switched between (A) pH 4.0 and 9.0 at 25 °C, (B) 25 and 40 °C at pH 9.0, (C) 0 M and 0.35 M Na_2SO_4 at pH 9.0 and at 25 °C and (D) 0% and 20% methanol at pH 9.0 and at 25 ºC.

Preparation and characterization of carboxylic multiwalled carbon nanotubes (c-MWCNTs)

First, carboxylic multiwalled carbon nanotubes (c-MWCNTs) were prepared using mixed acid oxidation. The original MWCNTs (0.5 g) were placed in a conical flask and added with 200 mL of mixed sulfuric acid and nitric acid (volume ratio of 3: 1). The mixed acid was placed in a bath at 60 °C and reacted for 4 h to form a homogeneous black solution. The solution was centrifuged to neutrality, and the product was dried in a vacuum oven at 100 °C for 8 h to obtain c-MWCNTs. Glassy carbon (GC) electrode was polished with 0.3 μm α-Al_2O_3 and then refreshed twice through ultrasonic cleaning in deionized water. Then, 2 μL 1 mg mL^{-1} c-MWCNTs–dimethylformamide suspensions was cast on the electrode surface and dried under infrared light to obtain c-MWCNTs/GC electrode.

The IR spectroscopy of MWCNTs and c-MWCNTs are shown in Figure S5. The stretching vibration peak of the carboxyl group ($\nu_{\text{-COOH}}$) of c-MWCNTs appeared at 1710 cm^{-1}, which indicates the existence of carboxyl group on the surface of MWCNTs [1]. On the other hand, the peak signal appeared at 1560 cm^{-1} in both IR spectra of as-synthesized and acid functionalized MWCNTs is associated to the carbon nanotubes backbone stretching mode. The presence of this peak gave an idea that the structure of MWCNTs was preserved after undergone acid treatment. Besides, a weak peak was appeared at 1400 cm^{-1} for acid functionalized MWCNTs which is associated to the O-H bending deformation mode of the carboxylic acid group[2]. The carboxyl group, which can improve dispersibility and solubility, was introduced on the surface after MWCNTs were treated with the mixed acid. The introduction of carboxylic acid groups was further demonstrated in transmission electron microscopy experiments. As seen in supplementary date Figures S6-A and S6-B that the surface of the c-MWCNTs is rough, with an observed “opening” phenomenon, and the terminal carbon atom is active and beneficial to the introduction of carboxylic acid groups.

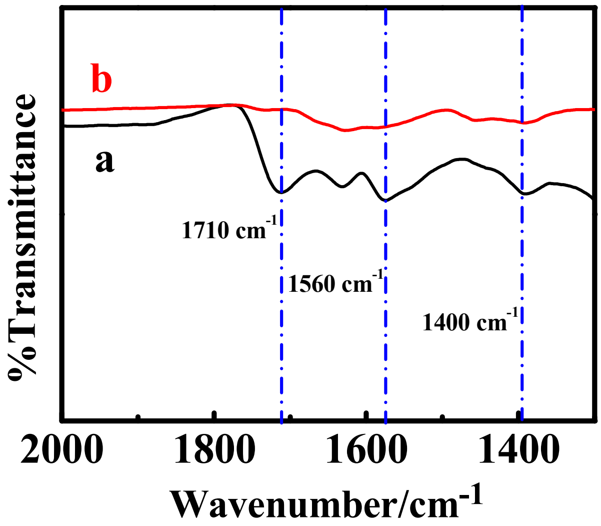

Figure S12 Fourier-transform infrared spectrum (FTIR) of (a) c-MWCNTs and (b) MWCNTs samples.

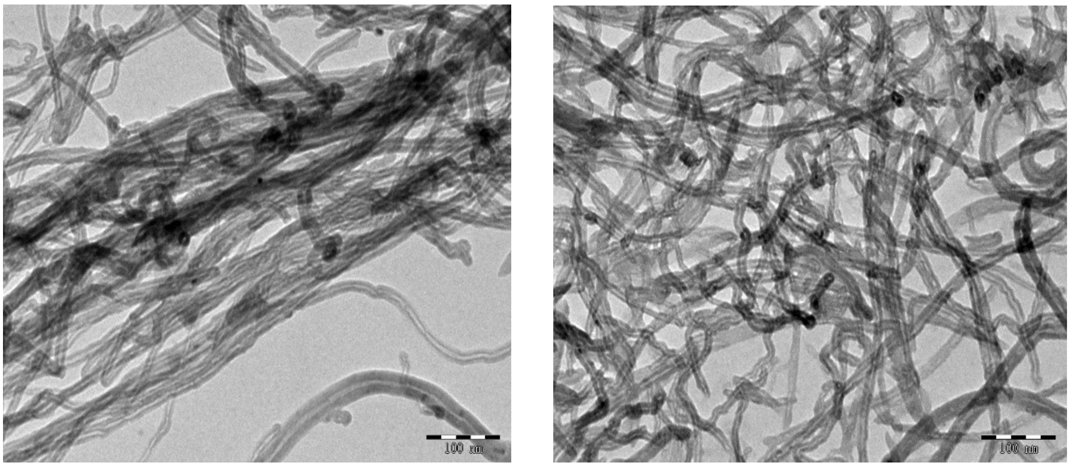

(a) **(b)**

Figure S13 Transmission electron microscopy (TEM) of (a) MWCNTs and (b) c-MWCNTs.

Table S1 Truth table

Inputs						Outputs
pH	Urease and urea	Temperature	Na_2SO_4	Methanol	Electrode type	I_{pa}
0	1	1	0	1	1	A
1	0	1	0	1	1	A
1	1	1	0	1	1	A
1	1	1	0	0	1	B
1	0	1	0	0	1	B
0	1	1	0	0	1	B
1	0	1	0	1	0	C
0	1	1	0	1	0	C
1	1	1	0	1	0	C
1	1	1	0	0	0	D
1	0	1	0	0	0	D
0	1	1	0	0	0	D
0	1	1	1	0	1	E
0	1	0	1	0	1	E
0	0	1	1	0	1	E
0	0	0	1	0	1	E
0	1	1	1	1	1	E
0	1	0	1	1	1	E
0	0	1	1	1	1	E
0	0	0	1	1	1	E
0	1	0	0	1	1	E
0	0	1	0	1	1	E
0	0	0	0	1	1	E
0	1	0	0	0	1	E
0	0	1	0	0	1	E
0	0	0	0	0	1	E
0	1	1	1	0	0	E
0	1	0	1	0	0	E
0	0	1	1	0	0	E
0	0	0	1	0	0	E
0	1	1	1	1	0	E
0	1	0	1	1	0	E
0	0	1	1	1	0	E
0	0	0	1	1	0	E
0	1	0	0	1	0	E
0	0	1	0	1	0	E
0	0	0	0	1	0	E
0	1	0	0	0	0	E
0	0	1	0	0	0	E

0	0	0	0	0	0	E
1	1	1	1	1	1	E
1	1	1	1	0	1	E
1	1	1	1	1	0	E
1	1	1	1	0	0	E
1	1	0	0	0	0	E
1	1	0	0	1	1	E
1	1	0	1	0	0	E
1	1	0	1	1	0	E
1	1	0	0	1	0	E
1	1	0	1	0	1	E
1	1	0	0	0	1	E
1	1	0	1	1	1	E
1	0	0	0	0	0	E
1	0	0	0	0	1	E
1	0	0	0	1	0	E
1	0	0	0	1	1	E
1	0	0	1	0	0	E
1	0	0	1	0	1	E
1	0	0	1	1	0	E
1	0	0	1	1	1	E
1	0	1	1	1	1	E
1	0	1	1	0	0	E
1	0	1	1	0	1	E
1	0	1	1	1	0	E

References

1. Deborah, M.; Jawahar, A.; Mathavan, T.; Dhas, M. K.; Benial, A. M., Spectroscopic studies on sidewall carboxylic acid functionalization of multi-walled carbon nanotubes with valine. *Spectrochim. Acta A Mol. Biomol. Spectrosc.* **2015,** *139*, 138-144.
2. Buanga, N. A.; Fadila , F.; Majida, Z. A.; shahirb, S., Characteristic of mild acid functionalized multiwalledcarbon nanotubes towards high dispersion with low structural defects. *Dig. J. Nanomater. Biostruct.* **2012,** *7*, 33-39.
